# Molybdenum Can Regulate the Expression of Molybdase Genes, Affect Molybdase Activity and Metabolites, and Promote the Cell Wall Bio-Synthesis of Tobacco Leaves

**DOI:** 10.3390/biology14010066

**Published:** 2025-01-14

**Authors:** Yuan Zhao, Yu Zhang, Kai Zhang, Jiashu Tian, Huanyu Teng, Zicheng Xu, Jiayang Xu, Huifang Shao, Wei Jia

**Affiliations:** 1 National Tobacco Cultivation and Physiology and Biochemistry Research Center, College of Tobacco Science, Henan Agricultural University, Zhengzhou 450002, China; zy18537717027@163.com (Y.Z.); 17527730075@163.com (Y.Z.); zhangkaikai233@163.com (K.Z.); jiashu0520@outlook.com (J.T.); 15737179601@163.com (H.T.); zichengxu@126.com (Z.X.); 2 College of Resources and Environment, Henan Agricultural University, Zhengzhou 450002, China; jiayangxu@henau.edu.cn

**Keywords:** molybdenum, metabolomics, molybdase, cell wall composition, FT-NIR spectroscopy, tobacco

## Abstract

Molybdenum (Mo) is widely used as a micronutrient fertilizer to improve plant growth and soil quality. However, the interactions between cell wall biosynthesis and molybdenum have not been explored sufficiently. This study thoroughly investigated the regulatory effects of different concentrations of Mo on tobacco cell wall biosynthesis from physiological and metabolomic aspects. The results indicate that Mo treatment increased the Mo content of tobacco variety K326. Moreover, it significantly up-regulated the gene expression levels of molybdases (*NR*, *AO*, *SO*, *XDH*) and molybdate transporters in tobacco, whereby the gene expression levels of NR were upregulated by 28.48%, 52.51%, 173.05%, and 246.21%, respectively; and MOT1 and MOT2 were upregulated by 21.49/8.67%, 66.05/30.44%, 93.05/93.26%, and 166.11/114.29%, respectively. Additionally, Mo treatment regulated the synthesis of related enzymes, effectively promoted plant growth, and significantly increased biomass and dry matter accumulation, with the biomass in the leaves increasing significantly by 30.73%, 40.72%, 46.34%, and 12.88%, respectively. The FT-NIR spectroscopy results indicate that after Mo was applied to the soil, the quantity of C-O-C, -COOH, C-H, and N-H functional groups increased. Concurrently, the contents of cellulose, hemicellulose, lignin, protopectin, and soluble pectin in the leaves significantly increased, wherein the content of soluble pectin and hemicellulose increased significantly by 31.01/288.82%, 40.69/343.43%, 69.93/241.73%, and 196.88/223.26%, respectively. Furthermore, the cell walls thickened, increasing the ability of the plant to withstand disturbances. The metabolic network diagrams indicate that Mo regulated galactose metabolism, and arginine and proline acid biosynthesis. The contents of carbohydrates, spermidine, proline, quinic acid, IAA, flavonoids, and other substances were increased, increasing the levels of polysaccharides and pectin within the cell wall, controlling lignin production, and successfully enhancing resistance to abiotic stress. These results offer important perspectives for further investigations into the role of trace elements.

## Simple Summary

Molybdenum (Mo) is an essential trace element for plant growth and development, and is currently widely used in crop fertilizers. The plant cell wall is the first barrier to prevent the invasion of diseases, and provide protection and support for plant growth. We systematically evaluated the regulatory effects of Mo on tobacco plant growth and cell wall biosynthesis. The application of Mo increased the Mo content, molybdase gene expression, and biomass, and thus promoted tobacco growth. The quantity of C-O-C, -COOH, C-H, and N-H functional groups, and the contents of cellulose, hemicellulose, lignin, protopectin, and soluble pectin were increased in the cell wall of Mo-treated plants. The metabolomics results revealed that Mo could regulate the synthesis of metabolic substances through key metabolic pathways such as galactose metabolism, and arginine and proline biosynthesis. Our findings reveal that Mo has beneficial effects on plant growth and cell wall thickening, and provides important clues for the further investigation of trace elements.

## 1. Introduction

Molybdenum (Mo), a vital trace element for plant growth and development, is instrumental in boosting antioxidant capacity, promoting carbon and nitrogen metabolism, and regulating hormones [1]. Mo is primarily taken up and transported in the form of molybdate [2]. It has no biological activity itself, but through complex biosynthetic mechanisms, it is integrated into the Mo cofactor (Moco) to create the active site of its associated enzyme, allowing it to perform its function [3]. Nitrate reductase (NR), sulfite oxidase (SO), aldehyde oxidase (AO), xanthine dehydrogenase (XDH), and mitochondrial-amidoxime-reducing component (mARC) are the primary molybdenum enzymes in plants that participate in nitrate assimilation, hormone biosynthesis, purine metabolism, sulfite detoxification, compound hydroxylation, and other bioecological processes [4]. Mo can influence carbon metabolism processes, increase the expression of genes associated with SPS and SPP enzymes, and notably increase both the net photosynthetic rate and photosynthetic pigment levels in tobacco [5]. Mo is essential for nitrogen metabolism and protein synthesis [6,7]. Mo serves as a cofactor for nitrogenase and nitrate reductase, the enzymes directly involved in nitrogen fixation and assimilation, thereby boosting efficient nitrogen metabolism [5]. Previous studies have demonstrated that Mo enhanced nitrate reductase activity in *Medicago sativa*, while Mo-based nanomaterials can improve root characteristics, promote nodule formation, and increase yield in leguminous crops such as soybean [8,9,10].

Mo regulates plant hormone and lipid levels, transmits signaling molecules, and maintains cell membrane integrity, thereby reducing plant damage [11]. AO can catalyze the production of IAA and ABA from indole-3-aldehyde and abscisic aldehyde. Therefore, under foliar molybdenum treatment, wheat plants show an increase in AO activity and a higher concentration of ABA [12,13]. Mo can increase the level of *CBF/DREB* genes, thereby increasing the content of free proline and phenolic compounds, and increasing resistance to Cd stress [14]. MoO_3_ nanoparticles (MoO_3_NPs) increase flavonoid levels, which, in turn, boosts nitrogenase activity and nitrogen content in soybeans [15]. Mo nanoparticles can enhance the nitrogen fixation ability of soybean plants by promoting the secretion of flavonoids and the expression of key genes [15]. Mo can increase the contents of linolenic acid (C18:3) and palmitoleic acid (C16:1) in wheat under cold stress [16], optimize the fatty acid composition in soybean, and increase the content of unsaturated lipids, particularly linoleic and linolenic acids, in glycerols [17].

The cell wall serves as a crucial barrier, forming the first line of defense against external substances and safeguarding cytoplasmic integrity. Furthermore, the cell wall has the unique capacity to adsorb, thereby bolstering plant resilience against abiotic stressors [18,19]. Lignin reinforces the cellulose structure, improving the mechanical strength of the plant and facilitating the transport of minerals through vascular bundles [20]. Studies have shown that Mo is involved in regulating the metabolism of wheat cell wall macromolecules and polysaccharides [21]. Mo application significantly increased the cellulose and hemicellulose contents in the winter wheat cultivar 97,014, helping maintain cell wall stability [11,16,21]. Under chromium stress, the combined application of Se and Mo increased the levels of pectin, cellulose, and lignin, enhancing both the crystallinity of the cell wall and the overall structure of the cell wall network [22].

Tobacco *(Nicotiana tabacum* L.), a member of the *Solanaceae* family, is a major model plant [23,24]. Due to its short growth cycle, well-characterized genetic background, and suitability for genetic manipulation, tobacco has become an important model organism. In this study, we conducted an in-depth investigation into the effects of molybdenum treatment on the physiological traits of tobacco, with a particular focus on its potential role in modulating cell wall composition, and we conducted metabolomics analysis to evaluate the physiological effects of molybdenum on tobacco growth. This comprehensive analysis highlights the molecular mechanism of molybdenum-induced changes in tobacco cell wall components at the metabolic level.

## 2. Materials and Methods

### 2.1. Pot Experiments

*N. tabacum* seeds (K326) were subjected to a series of treatments before germination. Seeds were initially soaked in a 10% (*v*/*v*) NaClO solution for 5 min, followed by a 30 s immersion in 70% (*v*/*v*) anhydrous ethanol. The seeds were then rinsed with sterile water and ultrapure water, and immersed for 8 h before germination on seed trays. After germination, the tobacco seedlings were transplanted into pots filled with vermiculite for a two-week period. The flower pot is a square box with a size of 7 × 7 cm, which can hold 100 g of soil. The soil type is loam. At this time, the yellow–brown earth soil received different treatments: CK (control), LT1 (0.1 mg kg^−1^ Mo), LT2 (0.2 mg kg^−1^ Mo), LT3 (0.4 mg kg^−1^ Mo), and LT4 (4 mg kg^−1^ Mo). Then, five-leaf-stage seedlings (50 days old after emergence) were transferred to treated soil for growth, with each treatment having six experimental replicates, and a total of three experiments were conducted. Mo was supplied in the form of Na_2_MoO_4_·2H_2_O, using an improved molybdenum-free Hoagland nutrient solution to fertilize plants and maintain plant growth. The formula is Ca(NO_3_)_2_·4H_2_O 945 mg L^−1^, KNO_3_ 607 mg L^−1^, NH_4_H_2_PO_4_ 115 mg L^−1^, MgSO_4_·7H_2_O 493 mg L^−1^, H_3_BO_3_ 2.86 mg L^−1^, MnCl_2_·4H_2_O 1.81 mg L^−1^, ZnSO_4_·7H_2_O 0.22 mg L^−1^, CuSO_4_·5H_2_O 0.08 mg L^−1^, FeSO_4_·7H_2_O 5.57 mg L^−1^, Na_2_-EDTA 7.45 mg L^−1^. The experiment was conducted at Henan Agricultural University in Zhengzhou, China. The temperature was maintained at 28/20 °C, the photoperiod was 14/10 h, and the relative humidity was 60–70%. After 28 days, tobacco plants in the seedling stage (78 days old after emergence) showed obvious symptoms. On the morning of the 30th day after treatment, the cleaned roots and 4th and 5th leaves of six different tobacco plants under each treatment were collected, marked and stored at −80 °C for physiological and biochemical parameter determination and gene expression analysis. The soil characteristics were as follows: pH, 8.14; organic matter content, 6.19 g/kg; total N, 23 mg/kg; available P, 4.10 mg/kg; available K, 277 mg/kg; and available Mo concentration, 0.05 mg/kg. Traditional agricultural practices were followed for other management measures.

### 2.2. Determination of Fresh Weight and Dry Weight

On the afternoon of the 29th day after treatment, cleaned roots and leaves were collected, the fresh weight of three different tobacco plants under each treatment was assessed, and the dry weight of the entire tobacco plant was measured after drying at 105 °C (0.5 h) and 70 °C, until a constant weight was reached. Three tobacco samples were tested in each treatment.

### 2.3. Fourier Transform Infrared Spectroscopy (FTIR) Analysis of the Cell Wall

Part of the roots and 4th and 5th leaves of 3 different tobacco plants collected from 9 a.m. to 10 a.m. on the 30th day after treatment was removed, followed by cell wall extraction. The cell walls of *N. tabacum* roots and shoots were blended with KBr (1:100 m/m), ground into fine dust, and pressed into thin flakes. The functional groups of the cell walls were measured by FTIR spectroscopy (Thermo Fisher Nicolet iS 50, Thermo Scientific, Waltham, MA, USA). A spectrometer (VERTEX 70) with a resolution of 4 cm^−1^ was used to record spectra in the range of 400 cm^−1^ to 4000 cm^−1^.

### 2.4. Assay of Cell Wall Components and Pectin Methylesterase Activity

Part of the 4th and 5th leaves of 3 different tobacco plants collected on the 30th day after treatment was used to determine the content of different components in the cell wall. Using the protopectin (BC3685) and soluble pectin (BC4125) test kits (Solarbio, Beijing, China), galacturonic acid was quantified via the carbazole sulfuric acid assay method [25]. The standard curve equation for galacturonic acid was y = 0.431x − 0.0256 (R^2^ = 0.9768), and the levels of protopectin and soluble pectin were calculated. The content of β-D-glucose was determined by the strong acid anthrone colorimetric method using cellulose (BC4285) and hemicellulose (BC4445) assay kits (Solarbio, Beijing, China) [26]. Hemicellulose levels were assessed using the 3,5-dinitrosalicylic acid (DNS) heating oxidation method [27]. The cellulose and hemicellulose levels were calculated from the standard curves for glucose (y = 8.3536x + 0.0388; R^2^ = 0.9703) and D-xylose (y = 5.2573x + 0.1673; R^2^ = 0.9972). The PE activity (BC2700) was determined using the NaOH titration method. The pH was adjusted to 7.8, and the reaction was accurately conducted in a 37 °C water bath for 60 min [28]. The amount of alkaline solution consumed to maintain a constant pH can reflect the PE activity [29]. The lignin content was quantified through the acetyl bromide method [30], with absorption readings taken at 280 nm and computations performed according to Bouguer–Lambert–Beer’s law: lignin = (mg/g) = ΔA ÷ ε ÷ d, ε = 23.35 mL mg^−1^ cm^−1^ and d = 1 cm [31].

### 2.5. Total RNA Extraction and qRT-PCR Analysis

Total RNA was extracted from the samples using a FastPure Universal Plant Total RNA Isolation Kit RC411 (Vazyme Biotech Co., Ltd., Shanghai, China). The extracted RNA was quantified, and its integrity was evaluated [32]. cDNA was synthesized using the HiScript III RT SuperMix kit and a gDNA eraser (Vazyme Biotech Co., Ltd., Shanghai, China). Real-time PCR was conducted with Taq Pro Universal SYBR qPCR Master Mix Q712 for precise quantification (Vazyme Biotech Co., Ltd., Shanghai, China) and TB Green™ (Takara, Tokyo, Japan) [33]. Supplementary Table S1 presents all primers utilized in the qPCR investigations, with L25 serving as the internal reference for relative expression calculations via the 2^−∆∆CT^ method [34].

### 2.6. Activity of Enzymes

The NR activity of the seedlings was assessed using a nitrate reductase assay kit (Solarbio, Beijing, China) following established protocols [35]. The enzyme-linked immunosorbent assay (ELISA) detection kits with serial numbers YJ845315, YJ845319, and YJ845316 (enzyme-linked immunosorbent assay, Shanghai) were used to measure the AO, SO, and XDH activities. The supernatant was added after grinding and centrifugation to the microplate coated with aldehyde oxidase antibody, and then bound with HRP-labeled aldehyde oxidase (AO) antibody to form an antibody–antigen–enzyme-labeled antibody complex. This was incubated at 37 °C for 60 min, then washed repeatedly 5 times. After that, the substrate TMB was added for color development, and finally, the stop solution was added. The method for measuring SO and XDH activity was the same as above. The absorbance (OD value) was measured at 450 nm, and the activity of AO, SO, and XDH calculated through different standard curves.

### 2.7. LC-MS Analysis

It was collected that the middle of the 4th and 5th leaves of 3 different tobacco plants on the morning of the 30th day after CK and LT2 treatments, respectively, and 6 replicates were set up. Approximately 60 mg of each sample was weighed and precooled at −40 °C for 2 min, ground at 60 Hz for 2 min (Wonbio-E, Shanghai, China), and transferred to a centrifuge tube containing 600 μL of a methanol/water solution (V:V = 7:3, containing a mixed internal standard at 4 μg/mL). After 30 min of ultrasonic extraction in an ice water bath, the mixture was allowed to stand overnight at −40 °C and then centrifuged at 12,000 rpm and 4 °C for 10 min. A total of 150 μL of the supernatant was filtered through a 0.22 μm organic-phase pinhole filter, and the filtrate was collected for LC-MS analysis.

An ultrahigh-performance liquid chromatography-mass spectrometry system consisting of Waters ACQUITY UPLC I-Class PLUS system (Waters Corporation, Milford, MA, USA) and Thermo QE instrument (Thermo Fisher Technologies, Waltham, MA, USA) was used for analysis. Two microliters of the sample was separated using a 100 mm × 2.1 mm ACQUITY UPLC HSS T3 1.8 μm column, and gradient elution was performed using a mobile phase consisting of A: water (containing 0.1% formic acid) and B: acetonitrile at a flow rate of 0.35 mL/min. The ESI source conditions were as follows: spray voltage, 3800 V to −3000 V; capillary temperature, 320 °C; auxiliary gas heater temperature, 350 °C; Gas1 temperature, 35 °C; and Gas2 temperature, 8 °C. The mass ranged from *m*/*z* 70 to 1050. The raw data were subjected to baseline filtering, peak identification, retention time correction, peak alignment, and normalization through Progenesis QI v3.0 software (Nonlinear Dynamics, Newcastle, UK), combined with MS-DIAL software for standardized preprocessing. Identification analysis was performed using the Human Metabolome Database (HMDB), Lipidmaps (v2.3), METLIN database, and LuMet Plant 3.0 local database.

### 2.8. Statistical Analysis

The experimental results are expressed as the mean ± standard deviation (SD). The statistical significance of the data was determined via Duncan’s test (*p* < 0.05) (SPSS 20.0). FTIR diagrams were generated using Origin 2018 software. GraphPad Prism 9.5.1 was used for data presentation.

## 3. Results

### 3.1. Regulatory Effects of Mo on Tobacco Growth Traits and Mo Content

**Figure 1:**
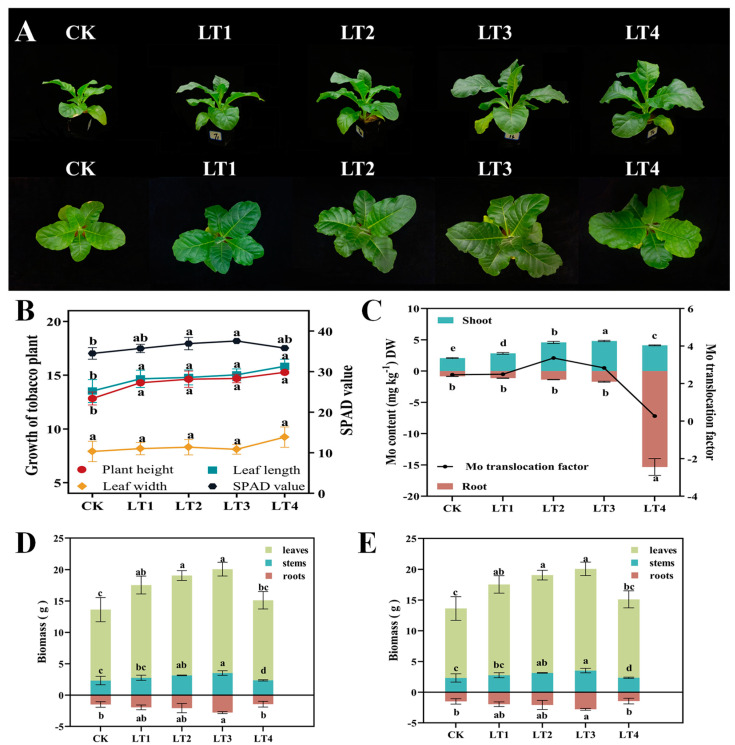
Regulatory effects of molybdenum on tobacco growth traits and Mo concentration. (**A**) Phenotypic pictures of tobacco plants; (**B**) growth of tobacco plants and SPAD value; (**C**) Mo concentration; (**D**) biomass; (**E**) dry matter accumulation. The values indicated by the line graph, bars graph, and error bars represent the mean ± SD (*n* = 3). Different lowercase letters denote statistically significant differences at *p* < 0.05.

Compared with CK, Mo treatment noticeably increased the plant height, leaf length, and SPAD value of tobacco seedlings, and the growth of the tobacco plants was significantly enhanced (Figure 1A,B). Within a certain concentration range, Mo treatment significantly increased the biomass and dry matter accumulation in the roots, stems, and leaves, and the aboveground molybdenum content, with the LT2 and LT3 treatments resulting in the greatest increase. When the Mo concentration reached 4 mg/kg, the biomass and dry matter accumulation in tobacco leaves decreased or were even slightly lower than those in CK, and slight chlorosis occurred in the tobacco leaves. Compared with the other treatments, the LT4 treatment significantly improved the underground molybdenum content (Figure 1C–E). Compared with those of the CK group, the plant height and leaf length of the treated tobacco seedlings significantly increased, but there was no substantial change in leaf width. The SPAD values of the tobacco plants significantly increased in response to the LT2 and LT3 treatments, with increases of 7.07% and 8.89%, respectively (Figure 1B). Furthermore, the LT2 and LT3 treatments significantly increased the biomass of the leaves and stems, by 40.72%/35.59% and 46.34%/51.70%, respectively. They also significantly increased the dry matter accumulation in leaves and stems, by 117.30%/75.19% and 145.07%/70.94%, respectively. Under LT4 treatment, neither factor caused a substantial change in other indices, except for a significant decrease in stem biomass (Figure 1D,E).

### 3.2. Effects of Mo on the Functional Groups and Ultrastructure of the Cell Wall in Tobacco Leaves

**Figure 2:**
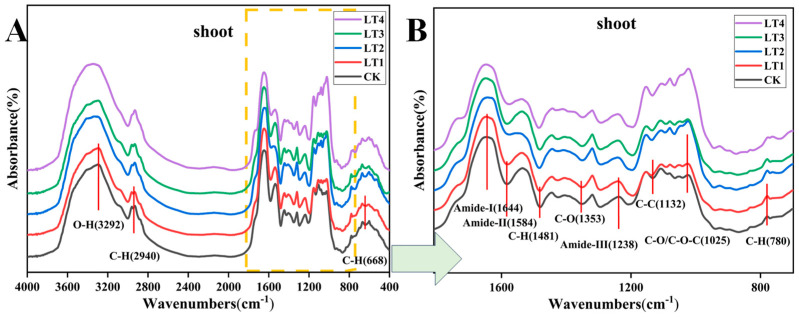
FTIR spectra of the cell walls of *N. tabacum* leaves under different molybdenum concentrations. (**A**) FTIR spectra of the *N. tabacum* cell wall in the 4000–500 cm^−1^ region under different Mo concentrations; (**B**) FTIR spectra of the *N. tabacum* cell wall in the 1800–700 cm^−1^ region under different Mo concentrations.

FT-NIR spectroscopy indicated that different concentrations of Mo affected the functional groups of the cell wall in tobacco leaves. Compared with the CK treatment, the absorbance of peaks associated with negatively charged functional groups (-OH, -CO, -CH, -NH, and -COOH) significantly increased with increasing Mo concentrations, with the COOH and -NH groups exhibiting the most significant changes. These results indicated that Mo increased the quantity of negatively charged moieties and influenced tobacco leaf composition by increasing the quantity of the components involved in cell wall formation. The spectral features at 3292 cm^−1^ and 2940 cm^−1^ for the leaf samples were associated with the OH/N-H and C-H groups, which are typically derived from proteins, cellulose, and pectin constituents [36] (Figure 2A, Table 1). The spectral range from 1700 to 700 cm^−1^ encompasses numerous distinctive bands associated with polysaccharides (such as pectin, cellulose, and hemicelluloses), lignin, amide functionalities, and esters. The primary vibrational peaks representing proteins are the absorption peaks of the amide I band (-NH) at 1644 cm^−1^ and the amide II band (-NH) at 1584 cm^−1^ [37], suggesting that the application of molybdenum could cause changes in cell wall proteins. According to previous reports, the spectral feature at 1353 cm^−1^ is indicative of the symmetrical stretching of COOH groups in pectin [38] and may also be related to the bending of CH_2_ groups in cellulose [39]. The change in the absorption peak at 1238 cm^−1^ may be related to changes in amide III or lignin structures [40]. The variations in the absorption peaks at 1132 cm^−1^ and 1025 cm^−1^ could be attributed to the C-O-C vibrations originating from cellulose and hemicellulose [41], or may be influenced by changes in lignin. The molybdenum treatments notably increased the intensity of the absorption peaks associated with the -CH and -NH groups, suggesting that the application of molybdenum induced changes in cell wall proteins (Figure 2B, Table 1).

### 3.3. Effect of Mo Application on Tobacco Cell Wall Composition

**Figure 3:**
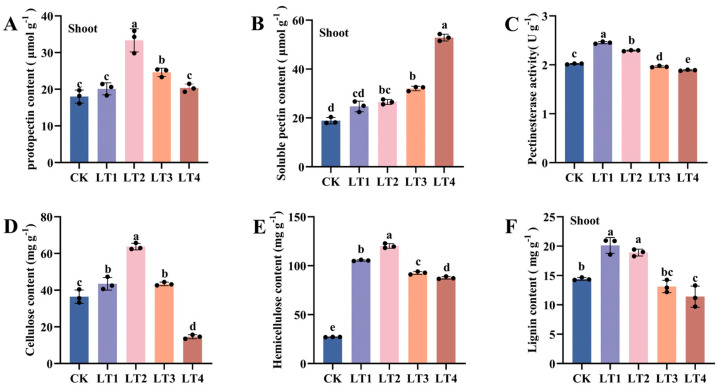
Effects of Mo concentration on the different composition contents and pectinesterase activity in *N. tabacum* cell walls. (**A**) Protopectin contents; (**B**) soluble pectin contents; (**C**); pectinesterase activity; (**D**) cellulose contents; (**E**) hemicellulose contents; (**F**) lignin contents. The values depicted by the bars represent the means ± SDs (*n* = 3). Different lowercase letters denote statistically significant differences at *p* < 0.05.

The application of Mo noticeably increased the contents of soluble pectin, protopectin, cellulose, hemicellulose, and lignin and pectin esterase (PE) activity in tobacco cell walls. Compared with the CK treatment, except for LT1 treatment, Mo treatment significantly increased the soluble pectin content of tobacco leaves by 40.69%, 69.93%, and 196.88% (Figure 3B). The contents of protopectin, cellulose, hemicellulose, and lignin and PE activity in tobacco leaves initially increased but then decreased. After Mo treatment, the hemicellulose content in tobacco leaves significantly increased by 288.8%, 343.4%, 241.7%, and 223.3% (Figure 3E). Under LT2 and LT3 treatment, the protopectin content of tobacco leaves increased by 85.20% and 36.74%, respectively, and the lignin content significantly increased by 39.71% and 31.21%, respectively (Figure 3A,F). The cellulose content in LT1, LT2, and LT3 increased significantly by 19.34%, 74.96%, and 19.14%, respectively. Under LT4 treatment, the cellulose and lignin contents were significantly reduced by 59.89% and 20.90%, respectively (Figure 3D–F). The PE activity of tobacco leaves significantly increased by 21.4% and 13.4% under LT1 and LT2 treatments, respectively, and significantly decreased by 2.8% and 6.4% under LT3 and LT4 treatments (Figure 3C).

### 3.4. Effects of Mo Application on the Relative Expression Levels of Genes Encoding Molybdenase-Containing Enzymes and Mo Transporters

**Figure 4:**
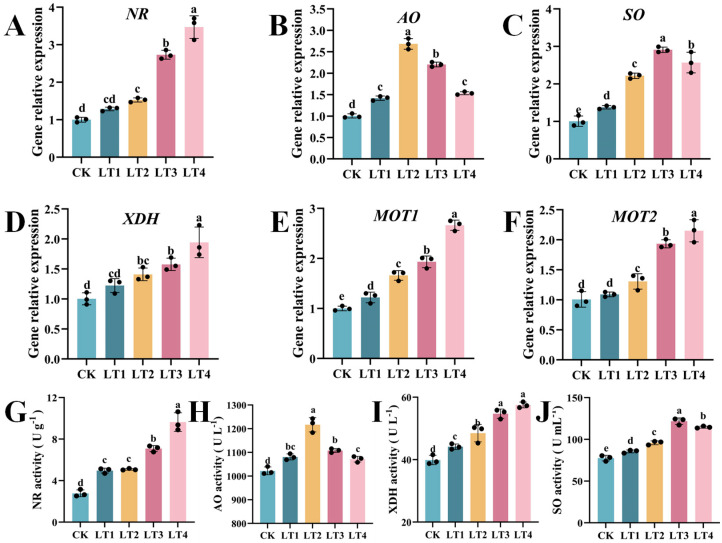
Effect of Mo concentration on the gene expression of the *N. tabacum* Mo enzyme and Mo transporter and on the activity of the Mo enzyme. (**A**) *NR* relative expression; (**B**) *AO* relative expression; (**C**) *SO* relative expression; (**D**) *XDH* relative expression; (**E**) *MOT1* relative expression; (**F**) *MOT2* relative expression; (**G**) NR activity; (**H**) AO activity; (**I**) XDH activity; (**J**) SO activity. The values depicted by the bars represent the means ± SDs (*n* = 3). Different lowercase letters denote statistically significant differences at *p* < 0.05.

By measuring the activity and relative expression levels of the genes *MOT1* and *MOT2*, which are involved in molybdenum transport in tobacco, as well as those of genes encoding four molybdenum-containing enzymes, NR, SO, AO, and XDH, the absorption and transport mechanisms of Mo within treatments with different concentrations of Mo were analyzed. The application of Mo notably increased the relative expression levels of *NR*, *SO*, *AO*, *XDH*, *MOT1,* and *MOT2*. Compared with CK, the expression levels of *NR* and *XDH* consistently increased with increasing molybdenum concentration. The LT3 and LT4 treatments significantly increased the expression levels of these genes by 173.05/57.28% and 246.21/93.19%, respectively (Figure 4A,D). Under LT2 treatment, the relative expression level of *AO* was the highest, with a significant increase by 168.20%, and then decreased, but it remained significantly lower than that under CK treatment (Figure 4B). Under LT3 treatment, the expression level of *SO* increased and reached its peak, significantly increasing by 190.79% before stabilizing (Figure 4C). Changes in Mo content strongly influenced the expression levels of the *NR*, *AO*, and *SO* genes. The expression levels of MOT1 and MOT2, two molybdate transporters, increase with increasing molybdenum concentrations. Under LT2, LT3, and LT4 treatments, significant upregulation by 66.05/30.44%, 93.05/93.26%, and 166.11/114.29%, respectively, was observed (Figure 4E,F). The gene expression level of molybdase affects the synthesis of the related enzymes. The findings of this investigation revealed that with an increasing molybdenum concentration, the NR and XDH activities significantly increased, and the AO and SO activities first increased but then decreased. Under the LT3 and LT4 treatments, the NR and XDH activities were increased by 155.47/37.19% and 247.21/44.19%, respectively (Figure 4G,J). The activity of AO was the highest under LT2 treatment, with a significant increase of 19.05%, while the activity of SO was the highest under LT3 treatment, with a significant increase of 57.04% (Figure 4H,I).

### 3.5. Metabolomics Analysis

**Figure 5:**
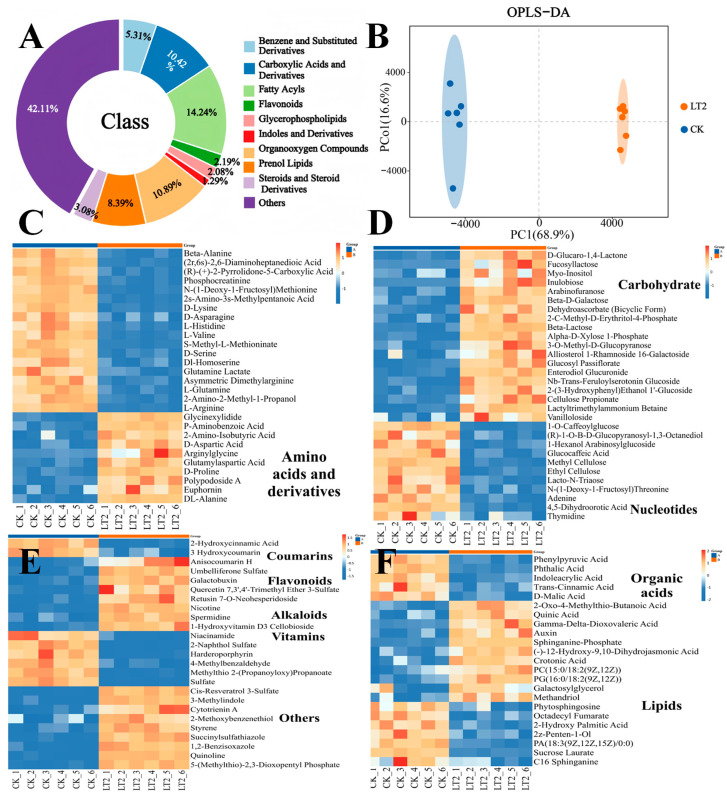
Metabolomics analysis of tobacco leaves under Mo treatment. (**A**) Classification of total differentially abundant metabolites; (**B**) score chart of OPLS–DA–generated differentially abundant metabolites. (**C–F**) Heatmap displaying the differential abundance of metabolites between the CK and LT2 samples.

On the basis of the above research results, we can conclude that the application of molybdenum resulted in a better growth of tobacco plants under LT2 treatment, and led to greater biomass and dry matter accumulation, increased cell wall component contents, elevated molybdenum enzyme activity, as well as increased gene expression levels. Therefore, we conducted an extensive LC-MS analysis of the target metabolite profiles of the CK and LT2 groups to investigate the metabolite changes in the tobacco variety K326 under molybdenum treatment. Principal component analysis (PCA) revealed that the first principal component accounted for 42.6% of the total variation, and the second principal component accounted for 22.4%, indicating reliable experimental results (Figure S1). The supervised orthogonal partial least squares-discriminant analysis (OPLS-DA) results showed that CK and LT2 samples were clustered on both sides of the x-axis (Figure 5B). Seven-fold cross-validation and tow-hundred-fold response permutation testing were applied, and the R^2^ and Q^2^ were 0.788 and −0.856, respectively (Figure S2), confirming that there were considerable variations in the metabolic spectra of tobacco leaves after different treatments without overfitting. The chemical classification and attribution information for each metabolite indicated that when undefined substances were excluded, the largest fatty acyl compounds accounted for 14.24% of the identified metabolites (Figure 5A). The next most-abundant compounds were carboxylic acids and their derivatives, as well as organic oxygen compounds, accounting for 10.89% and 10.42%, respectively. Using VIP > 1.5, FC > 1.2, or FC < 0.83, with a *p*-value < 0.05 as the standard, 107 significantly different metabolites were screened and represented in categorical clustering heatmaps (Figure 5C–F). Under LT2 treatment, carbohydrates (including fucosyllactose, myo-inositol, β-D-galactose, arabinofuranose, α-D-xylose-1-phosphate, β-lactose, methyl-D-erythritol phosphate, 3-O-methyl-D-pyranose, and cellulose propionate), lipids (including 12-hydroxy-9,10-dihydrojasmonic acid, octadecyl fumarate, and sucrose laurate), organic acids (including quinic acid, γ-Δ-dioxolanic acid, and auxin), flavonoids (galactobuxin and quercetin-7,3′,4′-trimethylether-3-sulfate), and alkaloids (nicotine and spermidine) significantly accumulated in tobacco leaves. The contents of amino acids and derivatives (including β-alanine, D-glutamic acid, L-isoleucine, D-lysine, L-histidine, L-valine, D-serine, Dl-homoserine, L-glutamine, and L-arginine) and nucleosides (adenine and thymidine) were decreased (Figure 5C–F). The variations in these metabolites offer valuable insights for investigating the effects of molybdenum on tobacco.

**Figure 6:**
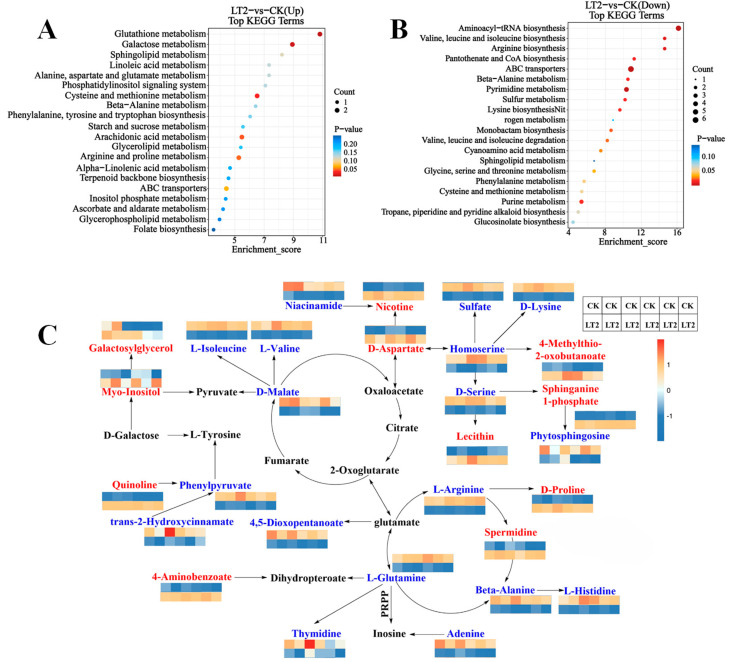
Differentially enriched metabolites in the metabolic pathways of *N. tabacum* leaves. (**A**,**B**) KEGG pathway enrichment bubble plot for the CK and LT2 samples. The bubble size and color intensity correspond proportionally to the pathway’s level of significance. (**C**) Metabolic network map based on different differentially abundant metabolites of the CK and LT2 samples.

To determine the main biological functions of the differentially abundant metabolites in the CK and LT2 groups, KEGG pathway enrichment analysis and metabolic network mapping were performed. The findings demonstrated that the main metabolic pathways related to Mo regulation included galactose metabolism, amino acid biosynthesis (arginine biosynthesis; arginine and proline metabolism; valine, leucine, and isoleucine biosynthesis; and β-alanine metabolism), glutathione metabolism, and pyrimidine metabolism (Figure 6A,B). With molybdenum application, a portion of D-malate participated in the galactose metabolism pathway, increasing the content of myo-inositol and galactosylglycerol. Another portion was used to convert and synthesize more D-aspartate acid, which was further converted into more nicotine through the nicotinic acid and nicotinamide metabolism pathways. L-arginine produced large amounts of spermidine and D-proline. The levels of β-alanine, L-histidine, and thymidine were downregulated through pathways such as beta-alanine and pyrimidine metabolism, and arginine and proline metabolism (Figure 6C).

## 4. Discussion

### 4.1. Mo Increased the Activities and Gene Expression of Mo-Related Enzymes, and Promoted the Growth Traits of Tobacco Plants

Molybdenum enzymes play crucial roles in vital plant processes; one such enzyme is nitrate reductase (NR), which facilitates the conversion of nitrate to nitrite, thereby enhancing nitrogen assimilation and optimizing its utilization efficiency [42]. Throughout this study, with the administration of Mo, the expression of genes encoding *NR*, *AO*, *SO*, and *XDH* in the leaves significantly increased, modifying the activity of NR, AO, SO, and XDH enzymes in the leaves, and increasing the ability of tobacco plants to withstand abiotic stress. As the Mo concentration increased, the activities of the NR, AO, SO, and XDH enzymes continued to increase until a concentration of 0.2 mg/kg was reached. Adding Mo can directly promote Moco generation and modulate the transcription of essential CNX proteins and Mo-dependent enzymes. *CNX6* is a cytoplasmic enzyme synthesized by Moco. An elevated expression of *OsCNX6* enhances the activities of NR, AO, XDH, and SO in rice [43]. Research has shown that Mo can regulate nitrogen metabolism, increase nitrate reductase activity, and thus improve plant nitrogen use efficiency [44], which is associated with effective nitrogen absorption and recovery at all Nf and Nr values [44]. Mo amplified the uptake of N-NO^3−^, promoted an increase in leaf nitrogen content and protein synthesis, and improved nitrate reductase activity in sugarcane [45], lemon balm leaves [46], maize, and soybean [9], which is consistent with the outcomes of this experimental study. Molybdenum nanofertilizer (MoS_2_NPs) substantially enhanced the transcription of genes encoding *AO* and *XDH* in soybean [10]. An increase in AO content can regulate the synthesis of the plant hormones ABA and IAA, stimulate tobacco plant growth. An increase in XDH content catalyzes the degradation of hypoxanthine into urate, enhances antioxidant capacity, and delays the aging process [47,48]. SO is located on peroxisomes [49], and it oxidizes sulfites and participates in the degradation of cysteine (Cys), which is a precursor of glutathione [50,51]. Therefore, an increase in SO content can protect plants from sulfite toxicity [52,53], regulate glutathione production, clear ROS, and reduce membrane damage. *ZmSO* can enhance drought resistance in maize [54] and tobacco by increasing the level of GSH-dependent antioxidants [55].

Molybdenum can regulate carbon metabolism and increase the net photosynthetic rate. An increase in NR activity can promote nitrogen use efficiency and protein synthesis, providing energy for tobacco plant growth. After the application of molybdenum, the plant height, leaf length, and SPAD value of the tobacco plants significantly increased, effectively promoting plant growth and significantly increasing the plant biomass, dry matter accumulation, and molybdenum content in the aboveground and underground parts. The optimal molybdenum concentration was 0.2 mg/kg. MOT1 and MOT2 are two high-affinity molybdenum transporters involved in molybdate absorption [53]. In the framework of this analysis, with increasing molybdenum concentrations, the expression of the *MOT1* and *MOT2* genes significantly increased. An increase in molybdenum transporter protein content is beneficial for the absorption, transport, and distribution of molybdate salts. This result was confirmed by the molybdenum contents in the roots and shoots of the tobacco plants. In the plasma membrane, MOT1 functions as the principal gateway for molybdate uptake in plant roots, enhancing the absorption of molybdate by root cells [56]. The transcription of *MOT1* depends on the presence of nitrate and is positively correlated with the expression level of NR [57]. MOT2 is globally expressed in leaf tissues, and an increase in *MOT2* expression can promote the distribution and migration of molybdenum between organs and the storage of excess molybdate salts in vacuoles [58].

### 4.2. Mo Increased Cell Wall Components and Thickened the Cell Wall Structure

The FTIR analysis of the functional groups in plant cell walls revealed that as the molybdenum concentration increased, the absorbance of multiple peaks corresponding to cell wall functional groups, such as pectin (-COOH), lignin (N-H), cellulose, and hemicellulose (C-O-C, C-C, C-H, O-H), in tobacco leaves increased significantly. More functional groups were detected, indicating that molybdenum application can promote the generation of pectin, polysaccharides, and lignin. The peak at 2940 cm^−1^ was attributed to the stretching of CH and CH_2_ in polysaccharides (mainly cellulose). The peak at 1644 cm^−1^ was attributed to the C¼O vibration of amide I in protein [37,59] or pectin esters [60]. The amide nitrogen (-NH) band in proteins undergoes deformation at approximately 1238 cm^−1^ [61]. The increase in absorbance of amide I band occurs at 1644 cm^−1^, amide II band at 1584 cm^−1^, and amide nitrogen (-NH) band at 1238 cm^−1^. The findings demonstrate that applying a higher concentration of molybdenum may lead to changes in cell wall proteins [62,63]. Of course, the peak at 1584 cm^−1^ may also be related to the asymmetric stretching vibration of the C=O in lignin [64] or attributed to the C=C stretching of HCA, as its structure is similar to lignin. According to previous reports, the spectral feature at 1353 cm^−1^ is indicative of the symmetrical stretching of COOH groups in pectin [38] and may also be related to the bending of CH_2_ groups in cellulose [39]. The peaks at 1132 cm^−1^ and 1025 cm^−1^ were attributed to the C-O vibration, which is usually coupled with a C-C vibration and mainly reflects the presence of cellulose [65,66], and also possibly attributed to C-O-C stretching from non-cellulose molecules such as arabinogalactan or pectin [67]. These observations imply that after molybdenum application, the number of COOH, C-O-C, C-H, and N-H groups increases, stimulating the synthesis of polysaccharides, lignin, and proteins, and thereby strengthening the mechanical properties of plant cell walls, enhancing the ability of tobacco plants.

This study demonstrated that Mo treatment notably enhanced the levels of protopectin, soluble pectin, cellulose, hemicellulose, and lignin in the leaves of the tobacco variety K326 during its growth period. This may stem from the potential of Mo to regulate the biosynthesis of pectin, cellulose, and hemicellulose. Under Mo treatment, the gene encoding UGP is upregulated, and UGP catalyzes the formation of UDP-Glc, which serves as a precursor for cellulose biosynthesis [22]. The UGE gene is upregulated, and through the NAD-driven redox alterations of UDP-GlcA, more UDP-GalA is produced, further catalyzing the accumulation of UDP-Xyl and UDP-Arap, providing pentose donors for hemicellulose synthesis [68]. The GAUT gene is upregulated, catalyzing the formation of more pectin from UDP-GalA. An increase in the cellulose content in tobacco cell walls, in conjunction with cell expansion and elongation, helps maintain the quantity of the cell wall matrix [69]. Hemicellulose is the predominant noncrystalline polysaccharide in plant cell walls, and interacts with cellulose and lignin to affect cell wall extension and elongation [69,70]. An increase in hemicellulose content can improve the stability of the cell wall network, regulate signaling molecules that control auxin-mediated growth expansion, promote cell growth, and improve plant tolerance. It has been reported that hemicellulose-1 (HC-1) in root cell walls can sequester approximately 68% of copper (Cu) in castor [41], combine with nearly 67% vanadium (V) in rice [66], and accumulate the majority of cadmium (Cd) and aluminum (Al) in Arabidopsis [71]. The increase in polysaccharide content in the cell wall may also be due to the participation of Mo in the chlorophyll biosynthesis pathway, passing through the epidermal layer, infiltrating the palisade and spongy mesophyll, and ultimately reaching the vascular bundle, thereby affecting stomatal conductance and increasing the net photosynthetic rate of plants [72]. It has also been demonstrated that the addition of molybdenum significantly increases the levels of cellulose and hemicellulose in the vegetative and blooming phases of wheat [21].

Moreover, the optimal concentration for Mo application was 0.2 mg/kg. At this level, the contents of pectin, cellulose, hemicellulose, and lignin and PE activity in the leaves are high. After this concentration threshold was exceeded, all indicators decreased, except for soluble pectin content, which decreased. In particular, at 4 mg/kg, the synthesis of cellulose and lignin was significantly inhibited, and a large amount of soluble pectin accumulated, with the content significantly increasing by 196.88%. This may be due the transport of excess Mo in the tobacco leaves, resulting in a substantial accumulation of soluble pectin. Carboxyl H in pectin effectively binds to MoO_4_^2−^ and restricts its transport to protoplasts, fixing it in the cell wall to sustain normal cellular processes [73]. Mo increased the contents of original pectin and soluble pectin. As the Mo concentration increased, the total pectin content markedly increased. With increasing Mo concentrations, the total pectin content significantly increased. Pectin fills the gaps between various microfiber filaments, which helps enhance cell adhesion and mechanical strength and improves plant tolerance. PE regulates the demethylation of pectin, converting original pectin into soluble pectin. In our study, PE activity increased at concentrations of 0.1–0.2 mg/kg Mo, possibly to coordinate the contents of original pectin and soluble pectin to modify the coagulation level of pectin gel and prevent the cell wall from becoming too hard and brittle. At concentrations of 0.4–4 mg/kg Mo, the PE activity decreased; this may be attributed to the elevated concentration of soluble pectin in the cell wall, which did not require further conversion. Studies have indicated that Mo inhibits PE activity and suppresses the conversion of original pectin to soluble pectin [21]. Lignin is formed through the polymerization of monolignin alcohols, which are biosynthesized from phenylalanine via the phenylpropanoid pathway. In this study, Mo induced lignin biosynthesis, with the greatest accumulation of lignin occurring at 0.1–0.2 mg/kg; this process enhanced the physical strength and permeability of biomass cell structures, thickened the cell walls, improved lodging resistance, and effectively resisted the influence of adverse external environments. The lignified cell wall endows plants with tolerance to drought and highly permeable environments, and is also an important barrier against pests and pathogens [74]. In cold-acclimated Rhododendron leaf tissues, an increased proportion of G and S lignin monomer units in the polymer structure enhances winter adaptation [75]. Lignin accumulation in the root endodermal cell wall may impede the transport of heavy metal ions into the xylem or prevent their efflux from the vascular bundle [76]. Prior studies have indicated that Mo can induce lignin biosynthesis; increase lignin accumulation; lead to the thickening of cell walls in rice [77] *Salix matsudana* [78] and *Brassica juncea* [79], and improve lodging resistance. However, at 4 mg/kg, the lignin content was lower than that of CK, which may be due to the formation of flavonoids through the phenylpropanoid pathway, which reduces the accumulation of monolignins and improves the ability of plants to neutralize free radicals and sequester metals [80]. Mo nanoparticles (NMos) may play a role in modulating phenylalanine metabolism, plant signal transduction, and Fla-R gene expression [15].

### 4.3. Mo Had Essential Effects on Carbohydrate, Amino Acid, and Secondary Metabolism

In total, 107 distinct metabolites were identified between tobacco samples treated with CK or LT2. These metabolites were mainly related to the metabolism of galactose; the biosynthesis of amino acids; and the biosynthetic and metabolic pathways of alkaloids, coumarins, and flavonoids. Myo-inositol is a product of galactose metabolism and is involved in cell wall formation, the phosphatidylinositol (PI) signaling pathway, and phytic acid biosynthesis [81,82,83]. Hemicellulose, a heterogeneous polysaccharide composed of hexose, pentose, and uronic acid, is typically measured by its total sugar content [84]. Pectin is an acidic heteropolysaccharide that is regulated by galactose metabolism. Pectin polysaccharides mainly include rhamnogalacturonan I, rhamnogalacturonan II, and high galacturonan, xylose, arabinogalactan, etc. [85]. After Mo treatment, the contents of fucosyllactose, α-D-xylose-1-phosphate, arabinofuranose, β-D-galactose, β-lactose, alliosterol-1-rhamnoside-16-galactose, 3-O-methyl-D-pyranose, enterodiol glucuronide, and vanilloloside in tobacco leaves were increased, providing sufficient substrates for the synthesis of more hemicellulose and pectin. Methyl cellulose, ethyl cellulose, and cellulose propionate, as cellulose derivatives, are closely related to the cellulose content. Thus, it can be deduced that Mo regulates carbon metabolism, influences pectin and polysaccharide levels in the cell wall, facilitates cell wall formation, and preserves cell wall functional stability, which aligns with the changes in polysaccharide and pectin contents observed under LT2 treatment in this study.

Myo-inositol stimulates the upregulation of the inositol galactoside synthase gene (*GolS*), strengthens abiotic stress resilience in alfalfa (*Medicago sativa* L.) hairy roots [86,87], enhances drought tolerance in MdMIPS1-overexpressing apple [82] and maize [88], and augments cold resistance in cucumber and tomato [89]. The soil application of Mo profoundly elevated the levels of phosphatidylcholine (PC), a primary cell membrane component critical for material transport and signal transmission, thereby mitigating cell membrane damage [11], which is consistent with our research. Fucosyllactose, myo-inositol, other sugar substances, galactosylglycerol, dehydroascorbate, and PC (15:0/18:2(9Z,12Z)), of which the levels increased in the tobacco plants, can serve as osmotic protectants, protecting the integrity of cell membranes and ensuring the normal operation of cells [90]. In parallel, the buildup of carbohydrate compounds supplies energy to support the growth of tobacco plants. The auxin and 3-methylindole contents were increased, promoting tobacco cell division and elongation [91], which aligns with the substantial increases in biomass and dry matter accumulation of tobacco plants under LT2 treatment in this study.

Spermidine ranks among the most prevalent polyamines (PAs) found in plants. PAs stimulate the de novo synthesis of antioxidant enzymes at the translational level, so spermidine functions as a plant growth regulator, intracellular messenger, and antioxidant [92]. Proline is synthesized in cytoplasmic sols and plastids, and is widely recognized as an important indicator of stress tolerance [90]. Nicotine typically originates from amino acid precursors converted through branched pathways [93,94]. According to the results of the present study, under Mo treatment, the level of lactyltrimethylammonium betaine increased, enhancing the ability of the plants to maintain cellular water balance [95]. An increase in D-aspartic acid content provides sufficient primers for the conversion and synthesis of nicotine. Increases in the spermidine and D-proline contents in leaves endow tobacco plants with higher antioxidant capacity, clears reactive oxygen species (ROS) (especially singlet oxygen), maintains cellular redox balance [96], protects cell structure, and effectively resists abiotic stress. It was reported that an increase in D-proline content enhanced drought tolerance in Egyptian wheat (*Triticum aestivum*) [97,98]. Exogenous Spd effectively increases antioxidant capacity, mitigates the overaccumulation of ROS, alleviates salinity–alkalinity stress in tomato [99] and cucumber [100], alleviates waterlogging-induced damage to maize seedlings [101] and enhances *Salix matsudana* tolerance to Pb [102]. L-arginine participates in the synthesis of D-proline and polyamines through the arginine and proline metabolism pathway. The amino-propyl group provided by S-adenosylmethionine (SAM) attaches to Put, sequentially synthesizing Spd and Spm [103]. Therefore, a decrease in the L-arginine content may drive increases in D-proline and spermidine levels through conversion. When the content of L-arginine decreases, the contents of β-alanine, L-histidine, thymidine, and L-glutamine are regulated through pathways such as beta-alanine metabolism and pyrimidine metabolism. On the other hand, the accumulation of peptides and proteins such as glycinexylidide, arginylglycine, glutamylaspartic acid, polysaccharide A, and euphornin can also contribute to a decrease in amino acid content.

Flavonoids can activate antioxidant enzymes, eliminate free radicals, and chelate metals, providing defense against abiotic stress [87,104]. P-coumaric acid, a precursor in flavonoid biosynthesis, is catalyzed by chalcone synthase (CHS) to form naringenin chalcone from p-coumaroyl-CoA and acetyl-CoA, which is subsequently converted into flavanones or naringenin by chalcone isomerase (CHI) [105]. Coumaroyl CoA can be converted to coumarol, which can polymerize into lignin, forming the H monomer of lignin [80]. This study demonstrated that after Mo treatment, the contents of flavonoids such as galactobuxin, quercetin 7,3′,4′-trimethyl ether 3-sulfate, and retusin 7-O-neohesperidosid were increased, indicating that the tobacco plants had increased stress resistance and defense effects. MoO_3_NPs enhanced the nitrogen-fixing capacity of soybean by stimulating flavonoid secretion and upregulating essential genes [15]. Quercetin 7,3′,4′-trimethyl ether 3-sulfate can regulate auxin transport through cellular transport and regulatory mechanisms [106]. Elevated levels of endogenous IAA improved cold tolerance in rapeseed [107], wheat [108], and peach [109]. Consistent with previous reports, higher auxin levels increased the resilience of tobacco plants to environmental stress [110,111]. It can be further inferred that the decreases in 2-hydroxycinnamic acid and 3-hydroxycoumarin contents in tobacco leaves may be related to the conversion of these compounds to flavonoids. Quinic acid, which participates in the shikimate pathway and is related to phenylpropanoid biosynthesis, modulates lignin accumulation in plants by regulating the expression of cinnamic acid hydroxylase (C4H) [112]. The findings of this study demonstrate that the increased quinic acid levels after the application of Mo provide a substrate for the synthesis of lignin, flavonoids, and anthocyanins [113]. Monolignans polymerize to form lignin, which enhances the hydrophobicity and rigidity of the vascular bundles in tobacco plants. The auxin signal is transduced by the *ARF* gene family, and research indicates that the PbARF19-driven auxin signaling pathway governs lignin biosynthesis in the stone cells of pear fruit [114] by regulating lignin biosynthesis genes. Therefore, it can be inferred that elevated lignin concentrations in the cell wall may also be regulated by auxin signaling.

## 5. Conclusions

We aimed to investigate the metabolic characteristics and physiological and biochemical properties of tobacco exposed to Mo to capture the comprehensive responses of the plant. The application of Mo promoted the biosynthesis of polysaccharides, lignin, and proteins in the leaf cell wall, thickened the cell wall, and promoted the resistance of tobacco plants to external stress. Simultaneously, the upregulation of *NR*, *AO*, *SO*, and *XDH* expression by Mo, along with the regulation of molybdase activity, promoted plant growth and dry matter accumulation. And the upregulation of *MOT1* and *MOT2* expression enhanced Mo absorption and transport. Furthermore, the metabolic analysis revealed that Mo significantly increased the levels of carbohydrates, flavonoids, spermidine, proline, quinic acid, and the plant hormone IAA, thereby regulating lignin biosynthesis.

## Figures and Tables

**Table 1:** This is a near-infrared spectroscopy data table.

Wavenumber	Functional Group	Absorbance				
		CK	LT1	LT2	LT3	LT4
780	C-H	0.1186	0.1203	0.1267	0.1101	0.1096
668	C-H	0.1965	0.1765	0.1864	0.1624	0.1805
1025	C-O/C-O-C	0.3696	0.3161	0.3686	0.3516	0.5243
1132	C-C	0.3507	0.2487	0.2798	0.2865	0.3655
1238	N-H	0.2373	0.2083	0.1904	0.2226	0.2510
1353	C-O	0.1952	0.1739	0.1791	0.1904	0.2358
1481	C-H	0.1746	0.1512	0.1415	0.1688	0.1726
1584	N-H	0.3152	0.3125	0.3089	0.3251	0.3188
1644	N-H	0.6003	0.6003	0.4855	0.5986	0.5513
2940	C-H	0.2798	0.2388	0.2306	0.2741	0.3298
3290	O-H	0.5200	0.4895	0.4437	0.5243	0.5918

## Data Availability

The data supporting this study’s findings are available in the Supplementary Figure S1 and Supplementary Table S1 of this article.

## Supplementary Materials

The following supporting information can be downloaded at: https://www.mdpi.com/article/10.3390/biology14010066/s1. Table.S1. Primer sequence used in this work. Figure S1. principal component analysis. Figure S2. 7-fold cross validation and 200 times response permutation testing.
