# Supplementary material for: Molybdenum Can Regulate the Expression of Molybdase Genes, Affect Molybdase Activity and Metabolites, and Promote the Cell Wall Bio-Synthesis of Tobacco Leaves
Source: Biology (Basel). 2025 Jan 14;14(1):66. doi: 10.3390/biology14010066 (PMC11762813; doi:10.3390/biology14010066)

SUPPORTING DATA:

Table.S1. Primer sequence used in this work.

Gene	No. ID	Primer sequences F (5' to 3')	Primer sequences R (5' to 3')
NR	XM_016650420.1	CTTAGCTTCTAGTCTCTGGTGA	CGTCTGCATAATGATATTCGT
AO	NM_001325428.1	AACACAATCCCAGTCCATCC	TTCCAACCTTCTCAATCCCTTC
SO	NM_001325307.1	CCCTGGAAGATGTGAGTGTTG	GTCTACCCTTTCTATGCCACG
XDH	XM_016618078.1	AGGAGAAGTTAGGGTAGGTGG	CATACTTCTGGTGCTTCTGAGG
MOT1	XM_016624068.1	ATCCTCATTTCCAAGCCCC	CCCATTGCACCATTTAGTTCAG
MOT2	XM_016590415.1	ACCTTTCCACCCTTCCATTC	GGGATGTAAGTGCCTAAGTCG
L25	L18908.1	CCGTCCAAAAAATCTGACCC	TCTTCAAAGTCTTAGGTCGG

Figure S1. 7-fold cross validation and 200 times response permutation testing.

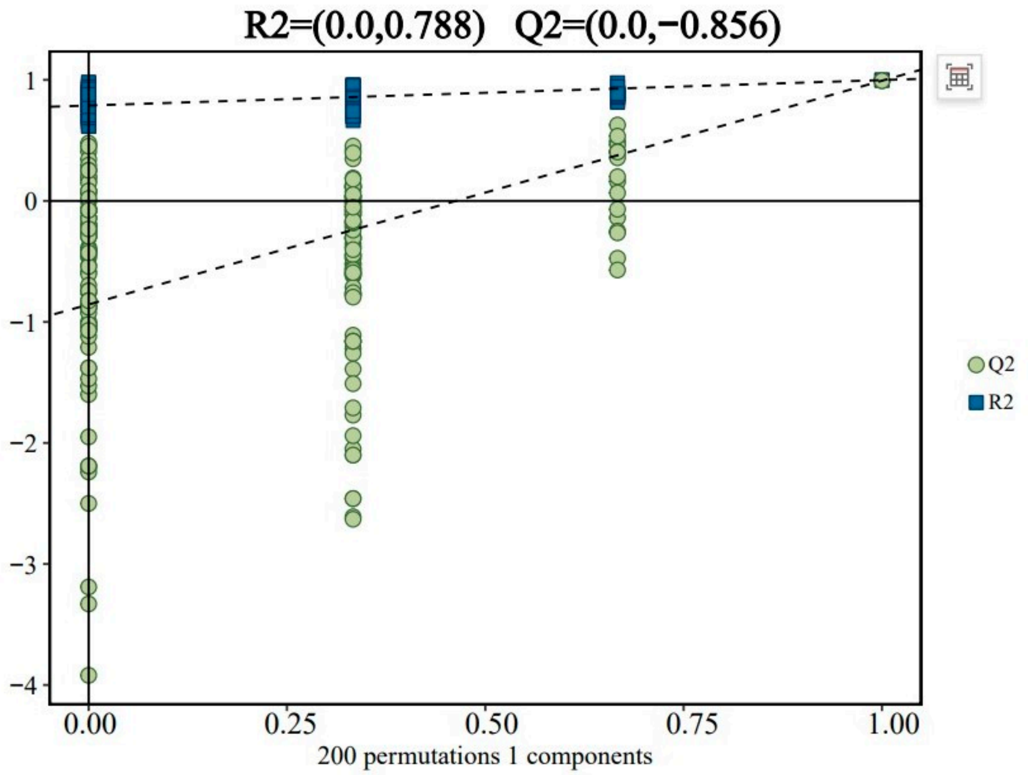

Supplement: Supplementary file 1 [file biology-14-00066-s001.zip › Table.S1.pdf]
