# Supplementary figures and images for: Molybdenum Can Regulate the Expression of Molybdase Genes, Affect Molybdase Activity and Metabolites, and Promote the Cell Wall Bio-Synthesis of Tobacco Leaves
Source: Biology (Basel). 2025 Jan 14;14(1):66. doi: 10.3390/biology14010066 (PMC11762813; doi:10.3390/biology14010066)

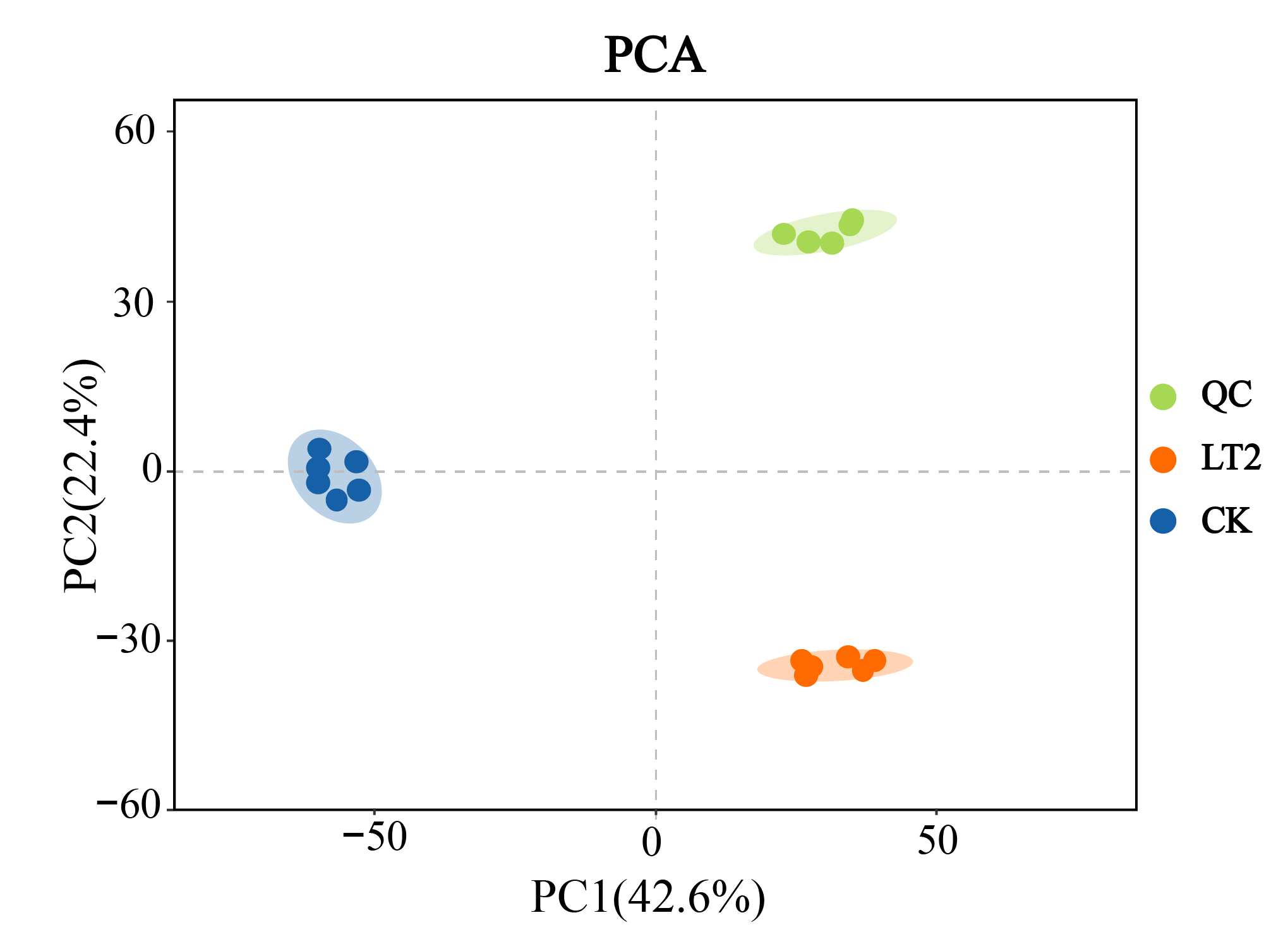

Supplement: Supplementary file 1 [file biology-14-00066-s001.zip › Figure S1.png]

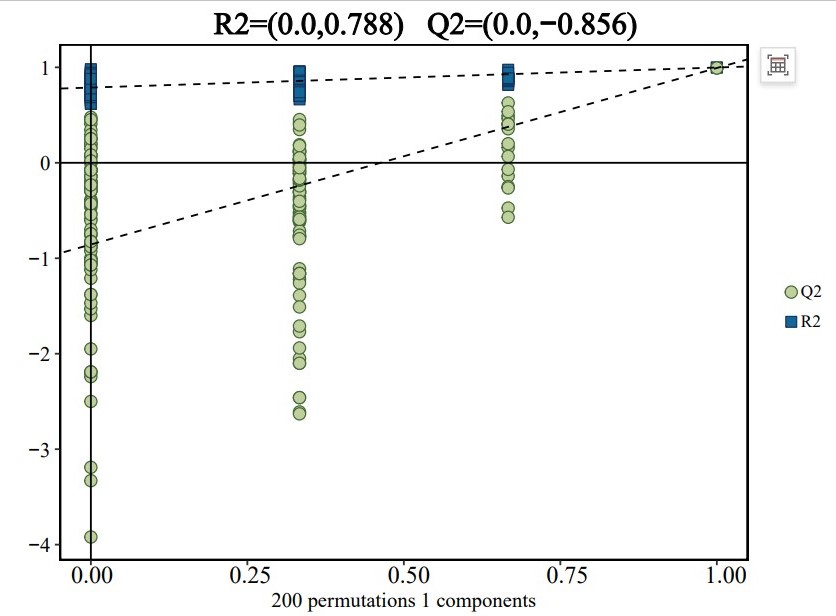

Supplement: Supplementary file 1 [file biology-14-00066-s001.zip › Figure S2.jpg]
